# Clinical application and observation of single-port inflatable mediastinoscopy combined with laparoscopy for radical esophagectomy in esophageal squamous cell carcinoma

**DOI:** 10.1186/s13019-020-01168-1

**Published:** 2020-06-05

**Authors:** Qifan Yin, Huining Liu, Yongbin Song, Shaohui Zhou, Guang Yang, Wenhao Wang, Peng Qie, Xuejiao Xun, Lijun Liu

**Affiliations:** Department of Thoracic Surgery, Hebei General Hospital, 348,West He-Ping Road, Shijiazhuang, 050051 Hebei Province People’s Republic of China

**Keywords:** Esophageal cancer, Non-transthoracic esophagectomy, Inflatable mediastinoscopy and laparoscopy

## Abstract

**Background:**

Transthoracic esophagectomy is a crucial independent risk factor for the incidence of postoperative cardiopulmonary complications in elderly or comorbid patients. To reduce postoperative cardiopulmonary complications and promote postoperative recovery. We made an attempt to adopt the single-port inflatable mediastinoscopy combined with laparoscopy for radical esophagectomy in esophageal cancer to observe the clinical application and effect.

**Method:**

Data of patients with esophageal carcinoma were collected in the Hebei General Hospital from May 2018 to November 2019. The operation time, surgical blood loss, the number of dissected lymph nodes, duration of drainage tube, duration of time on the ventilator, the length of stay in ICU, postoperative complications, the length of postoperative hospital stay were collected to assess the safety and feasibility of the single-port inflatable mediastinoscopy combined with laparoscopy for radical esophagectomy in esophageal cancer.

**Results:**

A total of 22 patients with esophageal cancer were analyzed in our research. There were no cases of conversion to thoracotomy、perioperative death or postoperative cardiopulmonary complications. The average operation time of all enrolled patients was 4.26 ± 0.52 h、The surgical blood loss was 142 ± 36.50 ml、The amount of dissected lymph nodes were 21.6 ± 4.2、The duration of drainage tube was 5.8 ± 2.5 days、The duration of time on the ventilator was 6.5 ± 3.4 h、The length of stay in ICU was 1.2 ± 0.4 days、The postoperative hospital stay was 12.6 ± 2.5 days. Among all the enrolled patients, one patient (4.5%) developed anastomotic fistula on the third day after surgery. Anastomotic stricture was found in 5 patients (22.7%). Pleural effusion was found in 4 cases (18.2%). Recurrent laryngeal nerve injury caused hoarseness or cough after drinking water in 3 cases (13.6%).There was one patient (4.5%) of conversion to laparotomy as the patient had serious peritoneal adhesion. All of the patients were discharged successfully.

Conclusion:Our results showed that this surgery of single-port inflatable mediastinoscopy combined with laparoscopy for radical esophagectomy in esophageal squamous cell carcinoma is safe and feasible. The feasibility and safety could be further and better investigated with a RCT to achieve more conclusive results.

## Introduction

Esophageal cancer is the sixth leading cause of cancer-related mortality and the eighth most common cancer worldwide, with a higher incidence in less developed and developing countries [1–4]. It has been reported that the incidence of esophageal cancer is the third highest and the mortality rate is the fourth highest among all cancers in china. More than half of global newly diagnosed esophageal cancer cases occur in China [5]. Esophageal cancer has a poor prognosis, The overall 5-year survival ranges from 15 to 25% [3, 4, 6]. Currently, Transthoracic esophagectomy has been the gold standard for potentially curable esophageal cancer [3]. However, it is associated with significant postoperative mortality and morbidity [7–9]. Pain and collapse of the lung (single-lung ventilation) due to thoracotomy may cause serious respiratory complications in elderly or comorbid patients. With the development of minimally invasive surgical technique, Thoracoscopic esophagectomy has been widely accepted as a minimally invasive surgery for esophageal cancer [10–12]. Nevertheless, in minimally invasive esophagectomy, the transthoracic approach, such as Ivor-Lewis esophagectomy and the three-incision modified McKeown esophagectomy, is most often adopted, Transthoracic surgery is a crucial independent risk factor for the incidence of postoperative pulmonary complications, [13] the main cause of morbidity and mortality following thoracotomy [14].

To reduce postoperative pulmonary and cardiac complications, considerable efforts have been made to develop the non-transthoracic esophagectomy for esophageal cancer. For instance, esophageal stripping and transhiatal esophagectomy are the non-transthoracic operations developed to cure esophageal cancer. These non-transthoracic esophagectomy possess several advantages, including non-thoracotomy, less postoperative pain, less postoperative cardiac and pulmonary complications, lower postoperative early mortality, and safer for elderly patients than transthoracic eaophagectomy. However, these methods are limited with the poor surgical view, poor mediastinal lymph node dissection (especially upper mediastinal lymph nodes), and high risk of bleeding [15, 16]. Therefore, non-transthoracic esophagectomy with effective dissection of mediastinal lymph nodes is challenging for radical resection of esophageal ccancer.

In 2015 and 2016, Prof. Fujiwara has developed novel surgical methods on the dissection of upper mediastinal lymph nodes using single-port mediastinoscopy through the cervical incision [17] and the lower mediastinal lymph nodes (including the subcarinal lymph nodes) by laparoscopy, respectively [18]. For the first time, non-transthoracic radical resection of esophageal cancer could be achieved along with the dissection of all the mediastinal lymph nodes. In oder to decrease postoperative cardiac and pulmonary complications, reduce postoperative early mortality, and promote recovery after surgery of elderly patients. Based on the Fujiwara’s method, we made an attempt to adopt single-port inflatable mediastinoscopy combined with laparoscopy for radical esophagectomy in esophageal cancer. We have completed 22 cases of radical resection of esophageal carcinoma using this novel surgical method from May 2018 to November 2019. Therefore, the purpose of this study was to report the clinical application and effect of this novel surgical method in these patients.

## Material and method.

### Patients

We had performed a total of 147 esophagestomy cases from May 2018 to November 2019 in our department in Hebei General Hospital. 22(15%) patients with esophageal cancer were enrolled into our research, all of whom had underwent single-port inflatable mediastinoscopy combined with laparoscopy for radical esophagectomy, 15 male and 7 female. The mean age was 65.5 ± 3.1 (range 58–72) years. The tumor location included upper thoracic (*n* = 5), middle thoracic (*n* = 13), and lower thoracic areas (*n* = 4). 12 patients had a history of smoking; The average BMI of enrolled patients was 23.12 ± 2.70 kg/m^2^;As for comorbidities, 9 patients with hypertension,7 patients with type 2 diabetes disease,6 patients with emphysema and 4 patients with coronary heart disease. The characteristics of the enrolled patients were shown in Table 1.

**Table 1:** The patients characteristics

Patients’ characteristics	Number of patients(*n* = 22)	Percentage(%)
Age		
Average	65.5 ± 3.1	
Range	58—72	
Gender		
Male	15	68.2%
Female	7	31.8%
Tumor location		
Upper thoracic	5	22.7%
Middle thoracic	13	59.1%
Lower thoracic	4	18.2%
Smoking status		
Smoker	12	45.5%
Non-smoker	10	54.5%
BMI	23.12 ± 2.70 kg/m^2^	
Comorbidity		
Hypertension		
Present	9	40.9%
Absent	13	59.1%
T2DM		
Present	7	31.8%
Absent	15	68.2%
Emphysema		
Present	6	27.3%
Absent	16	72.7%
Coronary heart disease		
Present	4	18.2%
Absent	18	81.8%

The inclusion criteria were as follows criteria: 1. Studied patients with esophageal squamous cell carcinoma were pathological examination confirmed; 2. The staging was limited to I-IIIA according to TNM classification of esophageal cancer as described in the 8th edition of the American Joint Committee on Cancer (AJCC); 3.The lesion did not exceed the muscular layer of the esophagus under endoscopic ultrasonography;4. The functions of the main organ system meeting the requirements of radical resection surgery; 5.patients did not receive neoadjuvant therapy; 6. Age of 18 to 75 years.

The exclusion criteria were; 1.The staging was IIIB or IV according to TNM classification;2. The patients merged with other cancers;3. Cervical esophageal squamous cell carcinoma;4.Patients with sereve cardiac、pulmonary、brain、renal complications cannot tolerate surgery; 5.Patients refused surgical treatment.

This study was approved by the institutional review board of our hospital and was conducted in accordance with the ethical principles of the Declaration of Helsinki. All patients provided written informed consent.

### Surgical procedure

**Fig. 1:**
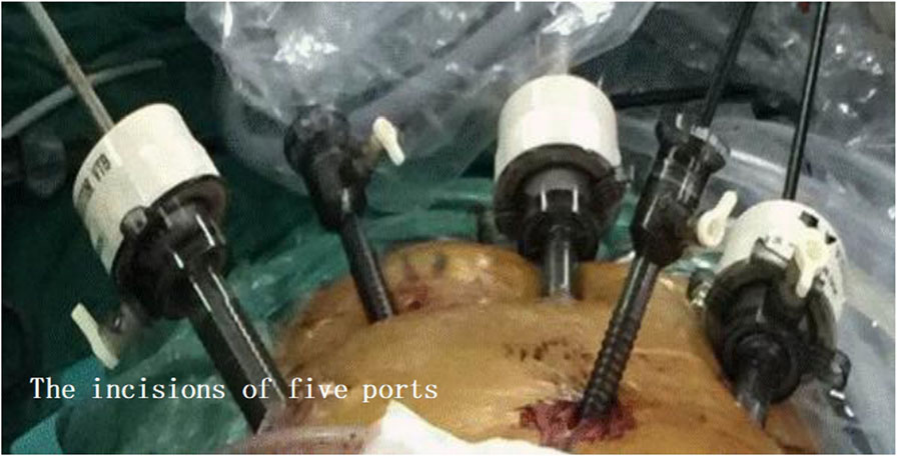
The incisions of five ports for the laparoscopic operation

**Fig. 2:**
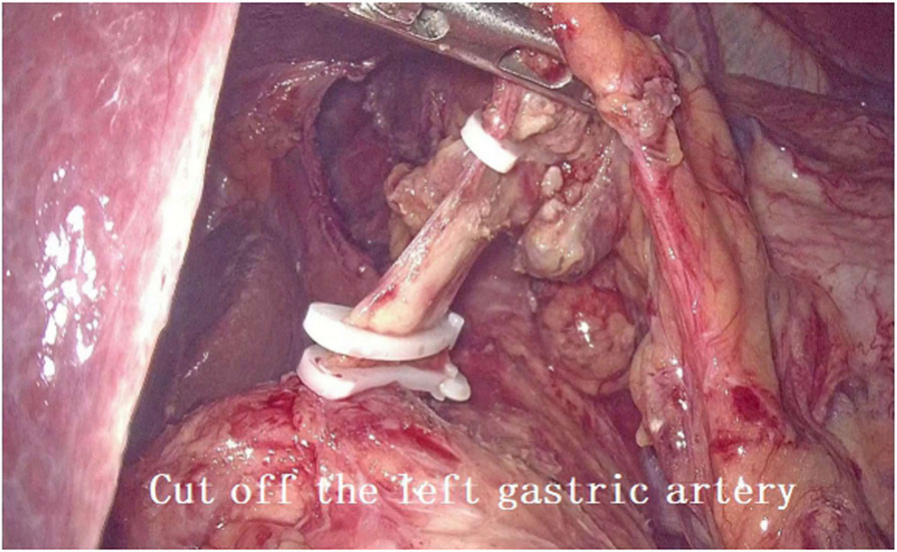
Cut off the left gastric artery

**Fig. 3:**
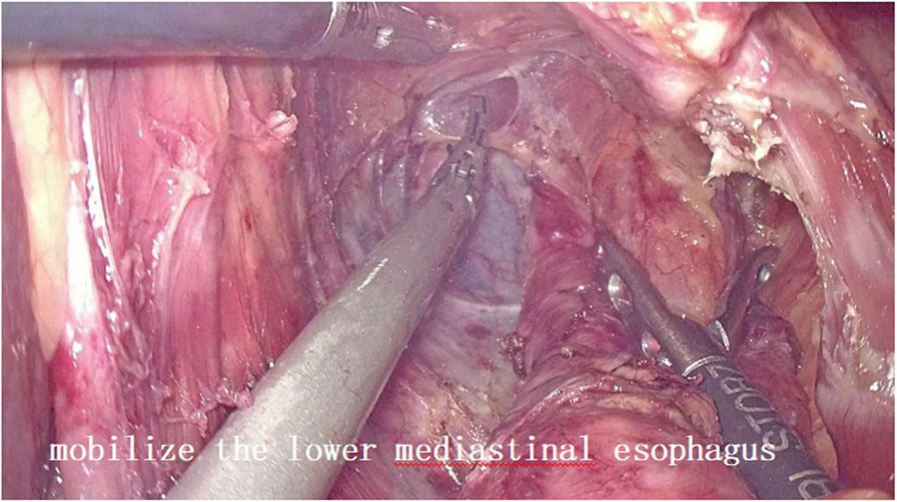
Moblize the lower mediastinal esophagus

**Fig. 4:**
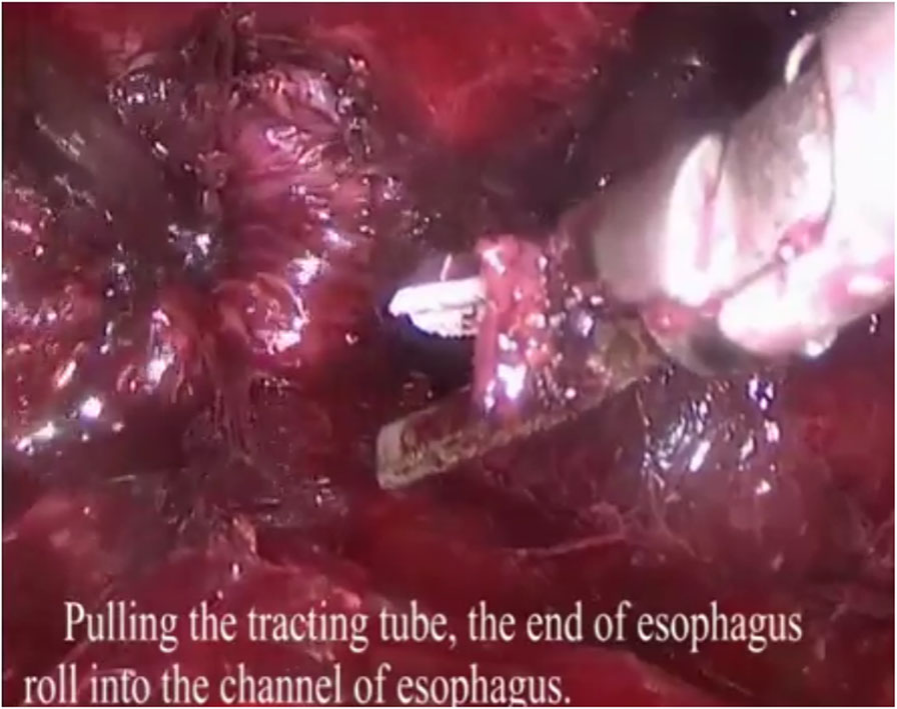
Pulling the tracting tube

**Fig. 5:**
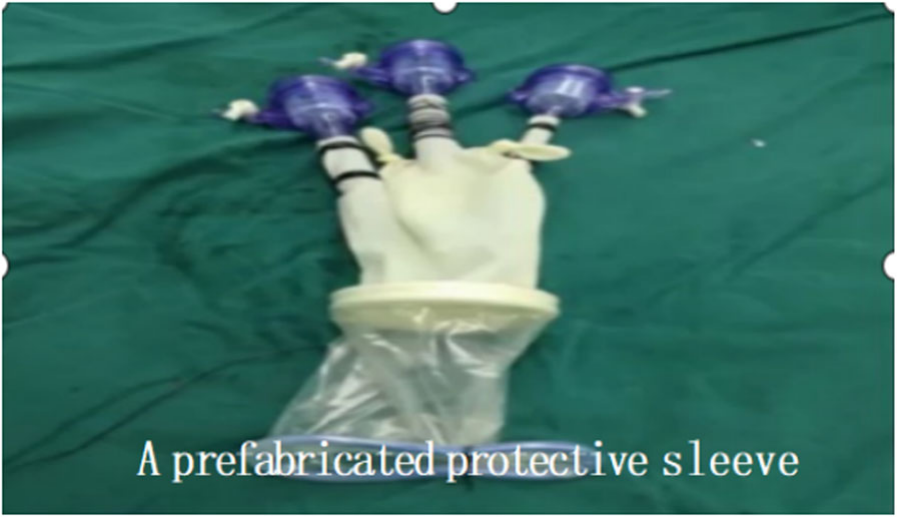
A prefabricated protective sleeve

**Fig. 6:**
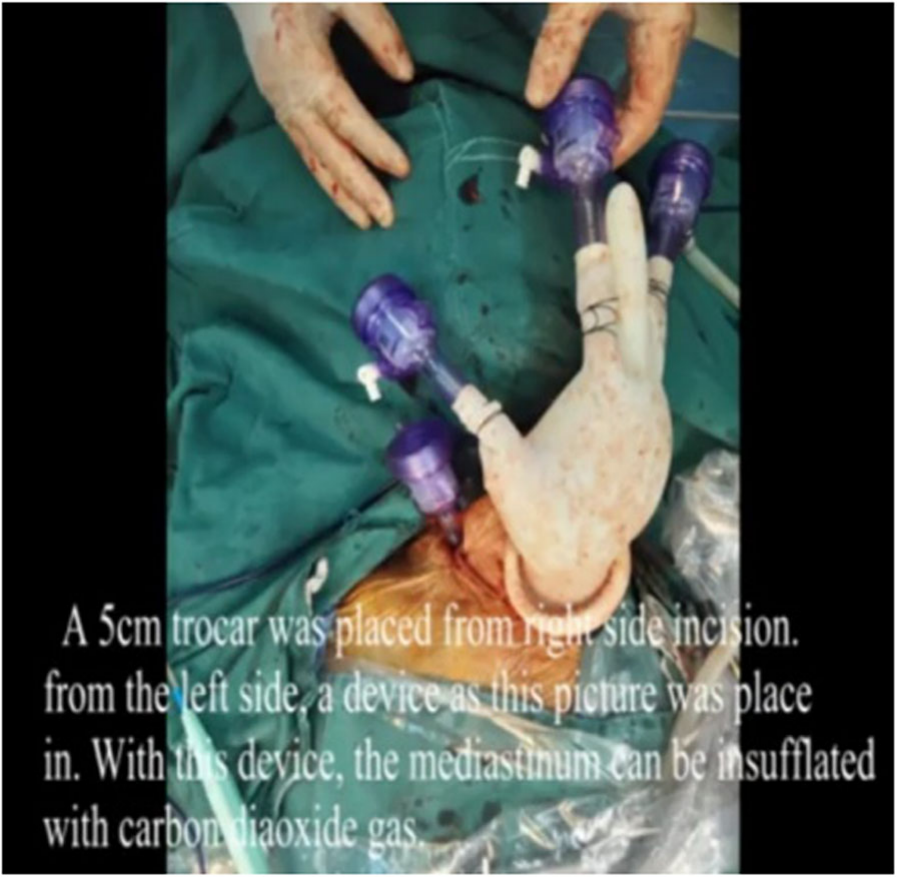
The cervical incision

1. Single lumen endotracheal tube was intubated after successfully anesthesia, Patients in the study were placed in the supine position, the upper abdomen and the neck were disinfected and drapped at the same time after properly fixed.

2. For abdominal operation: The puncture point was made at 1-cm above the umbilicus, establish artificial pneumoperitoneum. The incisions of five ports for the laparoscopic operation were made as follows: One 2-cm incision was made exteriorly at 1-cm above the umbilicus and used as the laparoscopic port, one 1-cm incision and one 5-mm incision were made at 3 cm from the paraumbilical region and used as the main operative ports. One 1-cm incision below the right costal margin and another 5-mm incision under xiphoid were made and used as the assisting ports (Fig. 1). Mobilization of stomach and abdominal lymphadenectomy were performed in the video-assisted laparoscopy using the conventional method (Fig. 2). Open the esophageal hiatus with an ultrasonic scalpel, dissect the lymph nodes around the cardia. We continued to mobilize the lower mediastinal esophagus along the esophagus until we reached the level of the carina or the ultrasonic scalpel cannot further reach (Fig. 3). Dissection of lymph nodes around the subcarinal and inferior pulmonary was performed successfully. A 5-cm subxiphoid vertical incision was made, through which the stomach was pulled out. Make a hole in the lesser curvature of stomach, through which the end of gastric tube that existed in the patient’s body before surgery was taken out, and then the end of this gastric tube was attached to the end of another tracting tube outside the body. The gastric tube junction was pulled into the stomach by anesthesiologist through the nose. The abdominal operation was over.

3. For left cervical operation: A 3–5 cm incision paralleling the anterior border of the sternocleidomastoid was made in the left neck, open the muscular layer. The left recurrent laryngeal nerve was identified and marked, The lymph nodes around the left recurrent laryngeal nerve were removed. First, The cervical esophagus was dissected and marked, the esophagus was mobilized with blunt dissection and cut at the leval of thoracic inlet. The gastric tube junction was be dragged to the distal end of esophagus by pulling the gastric tube. The junction of the two gastric tube was untied, and the end of the tracting tube that existed in the stomach and esophagus was fixed to the cutting edge of distal esophagus. Pulling the tracting tube, the cutting edge was enrolled into the channel of distal esophagus (Fig. 4). A pouch was made in the proximal end of the esophagus. A nail anvil was inserted into the pouch, and then the purse-string suture was tightened and fixed in preparation of anastomosis. A prefabricated protective sleeve with three trocars was inserted into the cervical incision (Fig. 5), and carbon dioxide was injected into the mediastinum with a pressure of 10 mmHg to cause artificial mediastinal emphysema. The assistant pulled the tracting tube in the abdomen to pull the distal end of the esophagus down, the distal end of esophagus turned inward and downward, while the operator mobilized the esophagus and the left recurrent laryngeal nerve in the neck. The blunt dissection was performed to resected the lymph nodes around the esophagus and the left recurrent laryngeal nerve, down to the lower edge of the aortic arch and the origin of left recurrent laryngeal nerve. During this procedure, attention should be paid to protect the azygos vein and tracheal membranous part. The esophagus was completely mobilized until the assistant pulled the esophagus with lesion into the abdominal cavity. The esophagus and stomach were taken out through the abdominal incision. Using cutting and stitching instruments, a tubular stomach with a width of about 4 cm was reconstructed. The tubular stomach attached with drainage tube was lifted up to the neck with a prefabricated long line through the esophageal bed of mediastinum. The distal end of drainage tube was separated from the tubular stomach. The stomach wall was cut at the highest point of the tubular stomach, and a circular stapler was inserted to anastomose the greater curvature side of the tubular stomach and the cervical esophageal. Then, a linear cutting stapler was used to close the proximal end of the tubular stomach at 2 cm from the anastomotic site. The gastric tube was inserted into the tubular stomach through the anastomsis. The left cervical incision was closed.

4. For the right cervical operation: A 1–2 cm incision paralleling the anterior border of the sternocleidomastoid was made in the right neck. The muscular layer was opened with blunt dissection, The trocar was inserted into the right cervical space as the main operative port. The left cervical incision was used as the assisting and endoscopic ports (Fig. 6). The right recurrent laryngeal nerve was identified and marked under the mediastinoscope, The lymph nodes around the right recurrent laryngeal nerve were removed. The right cervical incision was closed.

### Postoperative data collection

The operation time、surgical blood loss、the number of dissected lymph nodes、duration of drainage tube、duration of time on the ventilator、the length of stay in ICU、postoperative complications、the length of postoperative hospital stay were collected by the third author.

### Statistical analysis

Continuous data were presented as the mean ± standard deviation (SD). Categorical data were expressed as number (percentage). All statistical analyses were performed using IBM SPSS Version 21 (SPSS Statistics v21, IBM Corporation, Somers, NY, USA).

## Result

All the enrolled patients successfully completed surgery, The mediastinoscopy was successfully performed in all 22 patients. There was no case of death during the operation, while one case was switched from laparoscopy to open surgery as the patient had serious peritoneal adhesion due to previous laparoscopic cholecystectomy. In all patients, the postoperative pathological examination showed no evidence of residual tumor at the surgical margin. The R0 resection rate was 100%. Data collection and statistical analysis were performed on all enrolled patients. All quantitative data are expressed as mean ± standard deviation (SD). The postoperative data of the enrolled patients were shown in Table 2.

**Table 2:** Postoperative data collection

Postoperative observed indicators	Data
Operation time	
Average	4.26 ± 0.52 h
Range	3.2—5.5 h
Surgical blood loss	
Average	142 ± 36.50 ml
Range	65—210 ml
The number of dissected lymph nodes	
Average	21.6 ± 4.2
Range	14—29
Duration of drainage tube	
Average	5.8 ± 2.5 days
Range	3—13 days
**Duration of time on the ventilator**	
**Average**	**6.5 ± 3.4 h**
**Range**	**0.5–18.4 h**
**The length of stay in ICU**	
**Average**	**1.2 ± 0.4 days**
**Range**	**0–2.6 days**
The length of postoperative hospital stay	
Average	12.6 ± 2.5 days
Range	8—17 days

Operation time: The average operation time of all included subjects was 4.26 ± 0.52 h, the minimum was 3.2 h and the maximum is 5.5 h.

Surgical blood loss: The average surgical blood loss of all enrolled patients was 142 ± 36.50 ml, the minimum amount of blood loss was 65 ml, the maximum amount of blood loss was 210 ml.

The number of dissected lymph nodes: In all enrolled patients, the average amount of dissected lymph nodes were 21.6 ± 4.2, the minimum was 14 and the maximum was 29.

Duration of drainage tube: The average duration of drainage tube of all enrolled patients was 5.8 ± 2.5 days, the minimum duration of drainage tube was 3 days, the maximum duration was 13 days.

Duration of time on the ventilator: The average duration of time on the ventilator of all enrolled patients was 6.5 ± 3.4 h, the minimum duration of time on the ventilator was 0.5 h, the maximum duration of time on the ventilator 18.4 h.

The length of stay in ICU: 8 patients (36.4%) were not transferred to ICU after operation. The mean length of stay in ICU of all enrolled patients was 1.2 ± 0.4 days, the minimum length of stay in ICU was 0 days, the maximum length of stay in ICU was 2.6 days.

The length of postoperative hospital stay: The mean postoperative hospital stay of all enrolled patients was 12.6 ± 2.5 days, the minimum postoperative hospital stay was 8 days, the maximum postoperative hospital stay was 17 days.

Postoperative complications: Among all the enrolled patients, one patient (4.5%) developed anastomotic fistula on the third day after surgery, which was resolved by adequate drainage and nutritional treatment. Anastomotic stricture was found in 5 patients (22.7%), 3 of them with moderate or serious stricture received endoscopic balloon dilatation. Pleural effusion was found in 4 cases (18.2%), which were treated with adequate chest tube drainage. Recurrent laryngeal nerve injury caused hoarseness or cough after drinking water in 3 cases (13.6%). All patients with recurrent laryngeal nerve injury gradually recovered within 1 to 2 months because the nerve injury was temporary and reversible. There was one patient (4.5%) of conversion to laparotomy from laparoscopy as the patient had serious peritoneal adhesion. There was no perioperative death or postoperative cardiopulmonary complications. Eventually, All of the patients were discharged successfully. Perioperative complications were shown in Table 3.

**Table 3:** Analysis of patients’ complications

Complications	*N* = patients(%)
Anastomotic fistula	1 (4.5%)
anastomotic stricture	5 (22.7%)
Pleural effusion	4 (18.2%)
Recurrent laryngeal nerve injury	3 (13.6%)
Switch to laparotomy	1 (4.5%)
Switch to thoracotomy	0 (0%)
Cardiopulmonary complications	0 (0%)
Perioperative death	0 (0%)

## Discussion

After decades of research progress,the operation methods of esophageal cancer include transhiatal esophagectomy, transthoracic approaches,such as Ivor-Lewis esophagectomy and McKeown esophagectomy, left thoracotomy and left thoracoabdominal approach [19–23]. However, different surgical methods have different advantages and disadvantages, which depends on the location of the tumour and the preference of the surgeon. The surgical method described in this study was improved based on the methods described by Prof. Fujiwara, [17, 18] Affiliated Hospital of Kyoto Prefectural University of Medicine, Japan. During the abdominal operation, Carbon dioxide was pumped into the abdominal cavity to maintain the pressure at about 10–12 mmHg, The stomach was dissected and the lymph nodes in the abdominal cavity were removed using the conventional method. After opening the esophageal hiatus with an ultrasonic scalpel, We continued to mobilize the lower mediastinal esophagus along the esophagus until we reached the level of the carina. The artificial pneumoperitoneum provided a clear surgical view in the posterior mediastinal operative field, markedly reducing the difficulty for the resection of the mediastinal lymph nodes and dissociation of the lower esophagus. During the cervical operation, The artificial mediastinal emphysema provided a clear surgical view in the resection of the mediastinal lymph nodes and dissociation of the upper esophagus. Especially in the mobilization of the left recurrent laryngeal nerve.

Compared to transthoracic surgery, non-transthoracic surgery can effectively relieve postoperative pain, accelerate postoperative recovery and reduce the perioperative cardiopulmonary complications, thus expanding the surgical indications in the patients unable to receive transthoracic surgery, such as severe thoracic adhesions, chest abnormalities, and poor cardiopulmonary function. From our results, It shown that non-transthoracic surgery, significantly reducing the surgical trauma of patients, has great advantages over transthoracic surgery.

During the operation, there are some issues which should be paid special attention. We used an assistant to pull the tracting tube in the abdomen to pull the distal end of the esophagus down, while the operator mobilized the esophagus in the neck. During the process of assistant pulling the tracting tube, the distal end of esophagus turned inward and downward. Then a space was created in the upper mediastinum. This method makes it easier to mobilize the esophagus. In addition, Identification of the bilateral recurrent laryngeal nerve via the neck incision is crucial for a successful surgery. Therefore, the lymph nodes around the bilateral recurrent laryngeal nerve could be easily removed. It should be avoided to excessively dissect and expose the left recurrent laryngeal nerve as the excessive dissection would cause nerve demyelination, thus leading to nerve damage and postoperative hoarseness. For the dissection of the right recurrent laryngeal nerve lymph nodes, a 1-2 cm right cervical incision is needed.

Some shortcomings of this novel surgical method should be pointed out. First, the surgical field is smaller in the mediastinum than in the thorax. Successful surgery in the narrow surgical field highly depends on the experience of the surgeon. We found that the operation was very difficult in the few cases, especially with larger tumor. It was easy to injure some important tissues causing bleeding. Secondly, this surgical method was started to be performed in May 2018,hence,it’s long-term prognosis was still unknown. In addition, this study was a retrospective study, and we did not include patients receiving other methods for esophagectomy as the control group. In the following study, a well-designed, randomized controlled trial should be conducted to comprehensively evaluate the therapeutic efficacy and safety of this new surgical method. All these limitations should be addressed in the following study.

## Conclusion

In conclusion, Our results showed that this surgery of single-port inflatable mediastinoscopy combined with laparoscopy for radical esophagectomy in esophageal squamous cell carcinoma is safe and feasible. The feasibility and safety could be further and better investigated with a RCT to achieve more conclusive results.

## Data Availability

The datasets used and analysed during the current study are available from the corresponding author on reasonable request.

## Abbreviations

BMI: Body Mass Index
T2DM: Type 2 Diabetes Mellitus
AJCC: American Joint Committee on Cancer
SD: Standard Deviation
RCT: Randomized Controlled Trail
